# Perturbations of the Straight Transmembrane α-Helical Structure of the Amyloid Precursor Protein Affect Its Processing by γ-Secretase*[Fn FN2]

**DOI:** 10.1074/jbc.M113.470781

**Published:** 2014-01-27

**Authors:** Thomas Lemmin, Mitko Dimitrov, Patrick C. Fraering, Matteo Dal Peraro

**Affiliations:** From the ‡ Institute of Bioengineering and; the ¶ Brain Mind Institute, School of Life Sciences, Ecole Polytechnique Fédérale de Lausanne, CH-1015 Lausanne, Switzerland and; the § Swiss Institute of Bioinformatics, 1015 Lausanne, Switzerland

**Keywords:** Amyloid, Amyloid Precursor Protein, Mass Spectrometry (MS), Molecular Dynamics, Secretases, electron paramagnetic resonance (EPR) spectroscopy, double electron-electron resonance (DEER), metadynamics

## Abstract

The amyloid precursor protein (APP) is a widely expressed type I transmembrane (TM) glycoprotein present at the neuronal synapse. The proteolytic cleavage by γ-secretase of its C-terminal fragment produces amyloid-β (Aβ) peptides of different lengths, the deposition of which is an early indicator of Alzheimer disease. At present, there is no consensus on the conformation of the APP-TM domain at the biological membrane. Although structures have been determined by NMR in detergent micelles, their conformation is markedly different. Here we show by using molecular simulations that the APP-TM region systematically prefers a straight α-helical conformation once embedded in a membrane bilayer. However, APP-TM is highly flexible, and its secondary structure is strongly influenced by the surrounding lipid environment, as when enclosed in detergent micelles. This behavior is confirmed when analyzing *in silico* the atomistic APP-TM population observed by residual dipolar couplings and double electron-electron resonance spectroscopy. These structural and dynamic features are critical in the proteolytic processing of APP by the γ-secretase enzyme, as suggested by a series of Gly^700^ mutants. Affecting the hydration and flexibility of APP-TM, these mutants invariantly show an increase in the production of Aβ38 compared with Aβ40 peptides, which is reminiscent of the effect of γ-secretase modulators inhibitors.

## Teaser

**Background:** Amyloid-β neurotoxicity depends on the specificity of the proteolytic cleavage of the amyloid precursor protein (APP) transmembrane domain.

**Results:** The APP transmembrane α-helix is straight in a biological membrane bilayer.

**Conclusion:** The flexibility of APP is key for adapting to the lipid environment and modulating proteolytic processing by γ-secretase.

**Significance:** The dynamic characterization of APP is expected to rationalize the design of γ-secretase modulators.

## Introduction

The aggregation of amyloid-β (Aβ)^2^ peptides and the formation of extracellular Aβ plaques in brain tissue have been associated with the neuropathological process of Alzheimer disease. Aβ is a 38–43-amino acid-long peptide (Αβ38–43) contained within the C-terminal fragment of the amyloid precursor protein (APP), a type I transmembrane (TM) glycoprotein. Aβ is produced by the sequential cleavage of APP by β- and γ-secretases. The β-secretase first cleaves the extracellular domain of APP, producing a 99-amino acid-long TM protein, referred to as APP-C99. Then the proteolytic cleavage of APP-C99 by the γ-secretase occurs within the TM domain, releasing the Aβ peptides.

The neurotoxicity of Aβ depends on the peptide length and therefore on the position within APP-C99 of the γ-secretase cleavage site. Moreover, in Alzheimer patients, the ratio of Aβ42 *versus* Aβ40 is significantly higher. Effort has therefore been invested into characterizing the structure and dynamics of APP-C99 and how this substrate would interact with γ-secretase. Mutations in or adjacent to the TM region have a drastic effect on cleavage at the γ-site. More than half of the mutations in APP causing early onset familial Alzheimer disease are found in its TM domain (1). Moreover, the interaction of the APP with the membrane environment, in particular with cholesterol, Cu^2+^, and Zn^2+^ metal ions, alters the structural and dynamical properties of the protein (2, 3).

**FIGURE 1.**
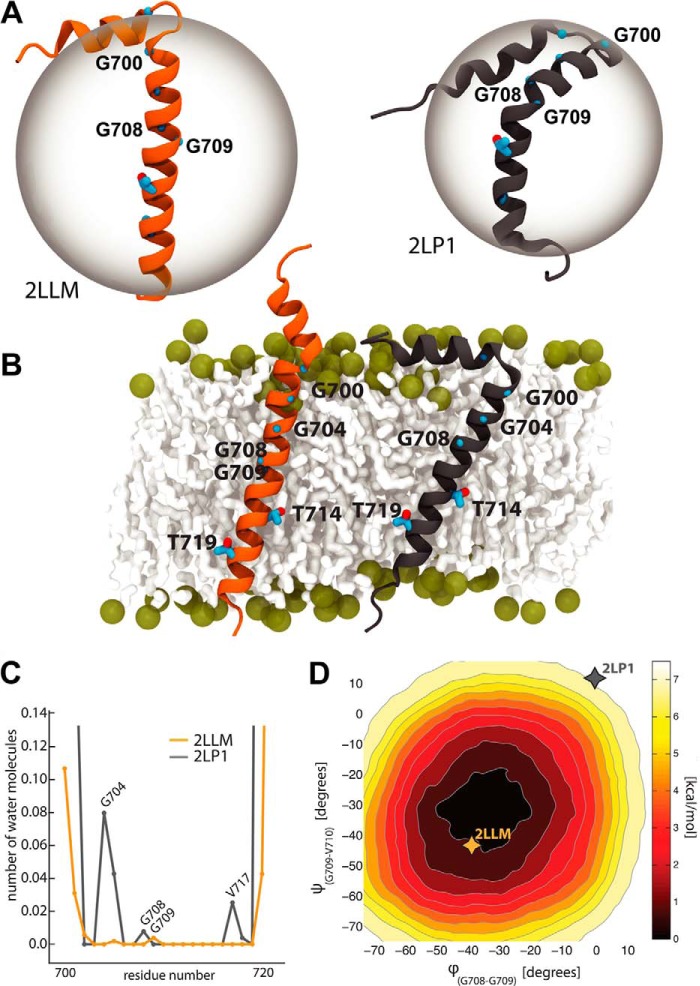
**Structure, dynamics, and hydration of APP-TM in a membrane bilayer.** *A*, NMR structures of the APP-TM domain (Protein Data Bank codes 2LLM (10) and 2LP1 (11)). The hypothetical micelle sizes inscribing the protein are displayed with *transparent spheres* and are based on the experimental setup reported in Refs. 10 and 11). Relevant glycine and threonine residues are shown in *blue. B*, representative MD snapshots of the NMR 2LLM (*left*) and 2LP1 (*right*) structures after ∼200 ns in a POPC bilayer. *C*, the relative level of hydration of each APP-TM residue for the two systems in the bilayer. *D*, free energy landscape associated to ϕ and ψ dihedral angles connecting the nitrogen atoms of Gly^708^ to Gly^709^ and Gly^709^ to Val^710^, respectively, as calculated by metadynamics MD. The average NMR conformations relative to the straight (2LLM [−41°; −46°]) and bent (2LP1 [−1°; 13°]) ensembles are reported with *orange* and *gray asterisks*, respectively.

Until recently, only a theoretical structure existed for the APP-TM domain. Miyashita *et al.* (4) used molecular dynamics (MD) simulations in implicit solvent to build and study a first atomic model of the APP-TM domain. They observed that Gly^708^ and Gly^709^ play an important role in the flexibility of the helix. This mechanism can stabilize the TM domain in the membrane, allowing it to adapt to the fluctuations of the membrane thickness. These residues are close to the γ-site and may directly influence substrate cleavage. The APP-TM contains two G*XXX*G motifs (*i.e.*, ^700^G*XXX*G*XXX*G^708^ in Fig. 1, *A* and *B*, and supplemental Fig. S1), which are believed to mediate dimerization in transmembrane proteins (5). Mutations of the G*XXX*G motifs also lead to a drastic decrease in the secretion of Aβ42 (6–8). MD simulations in implicit solvent showed that these mutations shifted the position of the γ-site in the membrane (9). Recently, however, two NMR structural ensembles have been solved. Nadezhdin *et al.* (10) determined the structure of APP-TM (Glu^686^–Lys^726^) in dodecylphosphocholine detergent micelles (Protein Data Bank code 2LLM; see Fig. 1*A*). The structures feature a short N terminus juxtamembrane helix (Lys^687^–Asp^694^) and a 24-amino acid TM helix (Gly^700^–Leu^723^). A minor bend (∼16°) was observed at residues Gly^708^-Gly^709^. These residues, located in the middle of the TM domain, can act as a hinge to compensate for the fluctuation of the membrane thickness.

Barrett *et al.* (11) more recently solved the NMR structure for the helical domain of APP-C99 (Val^683^–Tyr^728^) in lysomyristoylphosphatidyl glycerol (LMPG) detergent micelles (Protein Data Bank code 2LP1; see Fig. 1*A*). The N and C termini are mainly unstructured, except for a short C terminus helix, whereas the TM domain is characterized by a highly curved α-helix (∼115° at Gly^708^-Gly^709^). To validate the biological relevance of this kink, double electron-electron resonance (DEER) electron paramagnetic resonance (EPR) spectroscopy was used to estimate the distance between the ends of the TM domain in lipid bilayers. Methanethiosulfonate (MTSL) spin labels were attached to residues Gly^700^ and Leu^723^, mutated beforehand to cysteines. The distances measured in the micelles and lipid vesicles were nearly identical, thus providing indirect evidence that APP will also be curved in lipid bilayers. Later, Pester *et al.* (12) investigated the backbone dynamics at APP-TM and the effect of dimerization using NMR and MD simulations. They observed a bending motion at the G*XXX*G motif and a decrease of the dynamics of the binding site upon dimerization. The MD simulations were, however, carried out starting from an ideal α-helix model and did not consider the juxtamembrane helix or NMR model structures.

Here we used all-atom MD simulations to explain the existence of these divergent structures (see Fig. 1*A*), to understand the effect of the different experimental conditions on the APP conformations, and to obtain detailed insights into the dynamic properties of APP. We found that the conformational space of APP is strongly influenced by the size of enclosing micelles used in NMR experiments and by perturbations introduced by specific mutations and spin labels used during EPR experiments. Our results support a conformation for the APP-TM domain that is mainly populated by straight α-helical structures when embedded in a biological membrane bilayer. This conformation shows flexibility at the Gly^708^-Gly^709^ region, which can explain the multiple conformations that have been observed under different experimental conditions. Importantly, this flexibility probably has functional implications for γ-secretase presentation during proteolytic processing. We show in fact by *in silico* and *in vitro* functional analyses that position 700 is crucial for modulating such flexibility, producing a remarkable increase of hydration and production of Aβ38 in a series of Gly^700^ mutants. The fact that the same position is targeted by γ-secretase modulators can have functional implications to understand their therapeutic mode of action.

## EXPERIMENTAL PROCEDURES

### 

#### 

##### Molecular Simulation and Modeling

MD simulations were used to characterize the structure of APP in micelles and membrane bilayers, under the effect of different experimental conditions. The NMR structures (Protein Data Bank code 2LLM (10) and 2LP1 (11)) were inserted and equilibrated in a 60 × 60 Å^2^ palmitoyloleoly phosphatidylcholine (POPC) patch (13). The second structure was also inserted into LMPG micelles of diameter ∼35 and ∼45 Å, respectively. To test the effect of the spin labels used for DEER spectroscopy, we built cysteine or MTSL bound to cysteine point mutations (CM) on the APP. In total, nine APP mutants (seven single mutations: G700C, G700CM, L723C, L723CM, G700A, G700L, and G700W, and two double mutations: G700C/L723C and G700CM/L723CM) were inserted into a 60 × 60 Å^2^ POPC membrane. All systems were solvated in a 15 Å padding water box, neutralized by the addition of NaCl at a concentration of 150 mm (supplemental Table S1).

All simulations were performed using NAMD 2.8 (14) engine, with the CHARMM27 force field (15), including CMAP corrections for the protein and CHARMM36 for the POPC membrane (16). The parameters of the MTSL spin label were extracted from the work of Sezer *et al.* (7). TIP3P water (17) parameterization was used to describe the water molecules. The periodic electrostatic interactions were computed using particle mesh Ewald summation with a grid spacing smaller than 1 Å. Constant temperature (300 or 318 K) was imposed by using Langevin dynamics (18) with a damping coefficient of 1.0 ps. Constant pressure of 1 atm was maintained with Langevin piston dynamics (19), a 200-fs decay period, and 50-fs time constant. All systems were equilibrated for 20 ns at 300 K. Free molecular dynamics were performed up to hundreds of ns with a 2-fs integration time step using the RATTLE algorithm applied to all bonds. All MD simulations, listed in supplemental Table S1, sum up to a cumulative simulation time of ∼2 μs.

**FIGURE 2.**
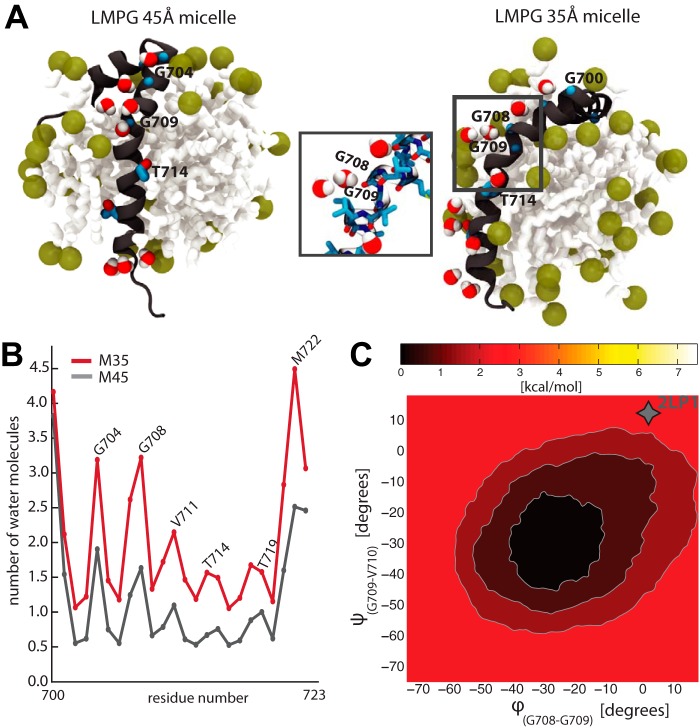
**Structure, dynamics, and hydration of APP-TM in detergent micelles.** *A*, representative MD snapshots of the APP_2LP1_ TM domain embedded into LMPG micelles of different sizes (35 and 45 Å, called M35 and M45, respectively) are shown. Glycine and threonine residues shown in blue are mainly exposed to the solvent. A focus of the Gly^708^-Gly^709^ region exposed to water molecules is reported in the *inset. B*, the distribution of the water molecules solvating the TM-APP tract is reported for the M45 and M35 systems. *C*, free energy landscape associated to ϕ and ψ dihedral angles as calculated by metadynamics MD on the M45 system (similar results obtained for M35 are shown in supplemental Fig. S3). The *gray asterisk* indicates the average NMR conformation relative to the 2LP1 ensembles.

**FIGURE 3.**
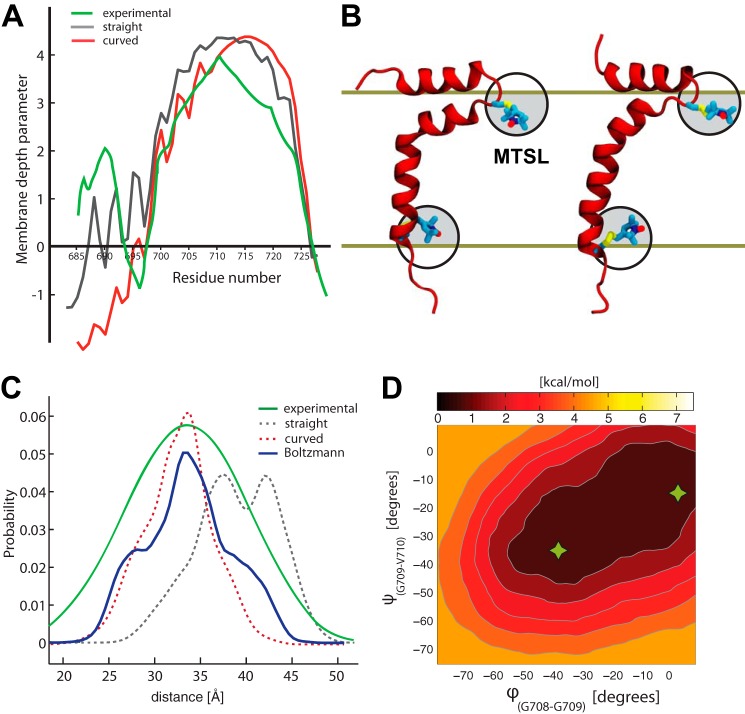
**Effects of EPR spin labels on APP structure and dynamics in the membrane.** *A*, the membrane depth parameter measured experimentally (11) (shown in *green*) is compared with the corresponding value computed from MD simulations for the straight and curved spin-labeled TM conformation in *black* and *red*, respectively. Positive values indicate burial into the membrane. *B*, representative MD snapshots of the MTSL spin labels attached to the curved (*left*) and straight (*right*) APP (a *solid line* indicates the average position of the membrane bilayer). The *black circles* outline the possible conformational space accessible to the MTSL in solution; because of the hydrophobic nature of the MTSL tag, the membrane restricts this conformational space (highlighted in *gray*). *C*, the MTSL distance distributions are compared as reported from DEER experiments (11) (in *green*), as predicted by using MMM for the MD ensembles of curved and straight conformations (in *red* and *black dashed lines*, other predictions are reported in supplemental Table S2), and as measured from unbiased and metadynamics MD simulations of the G700CM/L723CM system (in *blue*). *D*, free energy landscape associated to ϕ and ψ dihedral angles as calculated by metadynamics MD for the G700CM/L723CM system. The average curved [0°; −20°] and straight [−40°; −37°] conformations shown in *B* are indicated by *green asterisks*.

Metadynamics is used to enhance the sampling of the conformational space and to retrieve the associated free energy landscape (20). Gaussians are added to the energy surface and force the system to escape from local minima. This technique requires *a priori* knowledge of the degrees of freedom relevant to a conformational change. In the current systems, the observed conformational change was described by means of two collective variables representing the dihedral angles ϕ and ψ, which connect the nitrogen atoms of Gly^708^-Gly^709^ and Gly^709^-Val^710^. These angles allow sampling the different kinked conformations observed for the TM domain in the NMR structures. A set of metadynamics simulations (supplemental Table S1) was performed using the collective variable module of NAMD (21). Gaussians of width 0.3 and weight 0.01 were inserted every 300 fs. The conformational space was sampled from −75 to 10° for dihedral angles ϕ and ψ (see Figs. 1*D*, 2*C*, and 3*D*).

The residual dipolar couplings (RDCs) were back-calculated based on the orientation of the backbone N–H bond vector. Singular value decomposition was used to compute the alignment tensor that would best map the experimental RDCs. First, the N–H vector was extracted from the APP_2LP1_ ensemble, and then RDCs were back-computed. A set of structures was then extracted from the MD trajectories performed for the micelle systems. The structures that would best fit the experimental RDCs were selected with a genetic algorithm. We finally clustered them using the Jarvis-Patrick clustering algorithm (22) (supplemental Fig. S2).

Three different EPR DEER prediction algorithms were considered to estimate the distance distribution profile for the tagged APP-TM domain, namely MMM (Multiscale Modeling of Macromolecules; ETH Zurich), mtsslWizzard (24), and Pronox (25). The DEER signal was computed for the straight structure equilibrated in a POPC bilayer and the curved NMR structure. A modified version of MMM was used to estimate the distance distribution based on the MTSL coordinates extracted from the MD and metadynamics simulations (Fig. 3*C* and supplemental Fig. S6).

##### Molecular Biology

The pET21b vector encoding the cDNAs of human APP-C99-His was used as a template for the generation by PCR amplification of APP-C100-His substrates described in this study (recombinant APP CTF substrates expressed in *Escherichia coli* as a fusion protein consisting of a Met for translation initiation, amino acids 597–695 of the 695-amino acid isoform of APP, and the His_6_ tag sequence). The constructs were expressed in BL21(DE3) cells induced with 1 mm isopropyl-β-d-thiogalactopyranoside overnight at optic density at 600 nm of 0.8. For the purification of APP-C100 substrates (*i.e.*, wt, G700A, G700L, and G700W), harvested bacteria were lysed with 10 mm Tris, pH 7.0, 150 mm NaCl, 1% Triton X-100, protease inhibitor mixture (Roche Applied Science) and passed through a high pressure homogenizer (Emulsiflex-C5; Avestin, Inc., Mannheim, Germany). The obtained lysates were spun down at 5000 × *g* for 30 min, and supernatants were incubated overnight with nickel-nitrilotriacetic acid-agarose beads (Invitrogen) at 4 °C. Bound proteins were eluted in 1% Nonidet P-40 containing 250 mm imidazole (pH 7.8), analyzed by Coomassie-stained SDS-PAGE, and quantified by BCA (Pierce). The normalization was validated by Western blotting.

##### γ-Secretase Activity Assays

Highly purified γ-secretase (26, 27) was solubilized in 0.2% (w/v) CHAPSO, 50 mm HEPES (pH 7.0), 150 mm NaCl, 5 mm MgCl_2_, and 5 mm CaCl_2_ and incubated at 37 °C for 4 h with 1 μm substrate, 0.1% (w/v) phosphocholine, and 0.025% (w/v) phosphoethanolamine. The resulting products, APP intracellular domain (AICD-His) and Aβ, were detected with C-terminal APP antibody (Sigma-Aldrich) for AICDs and anti-Aβ antibody 6E10 (Covance, Berkeley, CA).

##### Immunoprecipitation-Mass Spectrometry Analysis of Aβ and AICD

**FIGURE 4.**
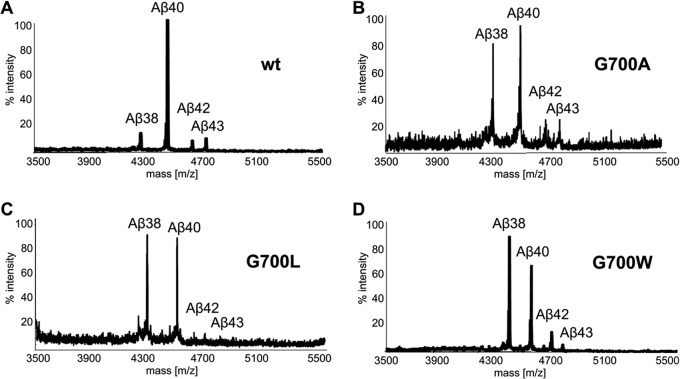
**Proteolytic processing of APP-C100 wild type and Gly^700^ mutants by purified γ-secretase.** Recombinant wild type (*A*) and mutant G700A (*B*), G700L (*C*), and G700W (*D*) APP-C100 substrates were expressed in *E. coli* and purified on a nickel-bound agarose column. After protein normalization, substrates were incubated for 4 h at 37 °C with highly purified γ-secretase, and the cleavage products Aβs and AICDs were immunoprecipitated and analyzed by mass spectrometry (IP/MS). *A–D*, IP/MS profiles revealed that Gly^700^ mutations alter APP γ-site-dependent Aβ products, by raising the relative quantity of Aβ38 without affecting the production of the longer and more toxic Aβ42 and Aβ43 peptides (see also supplemental Fig. S8*c* for ELISA quantification of Aβ38/Aβ40 ratios, and supplemental Fig. S8*b* for APP ϵ-site products AICD50 and AICD51). These experiments were performed twice, producing very comparable peak height values for each APP species.

Aβ and AICD generated in γ-secretase *in vitro* assays with purified substrates were analyzed as previously described (28). Briefly, Aβ peptides were immunoprecipitated overnight using anti-Aβ antibody 4G8 (Covance) and protein G-coupled to agarose resin (Roche Applied Science). For AICD detection, 1% Triton X-100 solution was added after the enzymatic reaction and incubated for 20 min at 55 °C on a shaker prior to overnight immunoprecipitation at 4 °C with protein A (Roche Applied Science), coupled with anti-C-terminal AICD antibody. Aβs and AICDs were eluted with 1:20:20 (v/v/v) 1% (v/v) trifluroacetic acid:acetonitrile:H_2_O, equally mixed with saturated α-cyano 4-hydroxy cinnaminic acid for detection of Aβs and synapic acid for AICD, and analyzed by MALDI-TOF mass spectrometry in reflectron mode on an ABI 4800 MALDI-TOF/TOF mass spectrometer (Applied Biosystems, Carlsbad, CA). Molecular masses were accurately measured and searched against amino acid sequences of human APP-C99 with the addition of a methionine residue at the N terminus and a His_6_ tag sequence at the C terminus (C100His). These experiments for each species were performed in duplicate with comparable observed peak height values (Fig. 4 and supplemental Fig. S8*b*).

##### Enzyme-linked Immunosorbent Assay

Aβ38 and Aβ40 peptides were quantified using human Aβ38 and Aβ40 commercially available ELISA kits from Invitrogen or IBL International (Hamburg, Germany), respectively. ELISA measurements (in triplicate for each APP species, see supplemental Fig. S8*c*) were performed according to the manufacturer's instructions.

## RESULTS

We carried out an extensive set of MD simulations of APP-TM structures considering the effect of a POPC lipid bilayer and LMPG micelles. We compared and validated our results with the available experimental data (namely, NMR and EPR (11)). We also tested the effects of the experimental protocols, for example those produced by spin labeling of APP. MD simulations of proteins in the membrane might require a long simulation time to reach equilibrium. Enhanced sampling techniques such as local elevation (29), conformational flooding (30), or metadynamics (20) have proven to be useful to efficiently explore the conformational space during molecular dynamics simulations. In this study, we used metadynamics, as successfully done for other membrane-spanning proteins (31), to investigate the dynamic determinants of the APP-TM domain and to reconstruct the corresponding free energy landscape under relevant external conditions. Finally, these results were used to rationally design and investigate a series of mutations affecting the key region found to modulate the flexibility of APP-TM, and their effect on the proteolytic processing by γ-secretase was tested *in vitro*.

### 

#### 

##### The APP-TM Domain Has a Predominantly Straight Helical Conformation in a Membrane Bilayer

We considered the two available NMR structural ensembles (Protein Data Bank codes 2LLM and 2LP1), referred to hereinafter as APP_2LP1_ and APP_2LLM_, respectively. APP_2LLM_ is solved as a mostly straight α-helix with a small concavity near Gly^708^-Gly^709^, whereas the APP_2LP1_ structure is characterized by an important kink at this region (Fig. 1*A*). To characterize these structures in a physiological-like environment, we inserted them into a membrane bilayer formed by POPC, which is a major lipid constituent of the synaptic plasma membrane (32). The MD simulations were carried out under physiological-like conditions (*e.g.*, pH ∼7, 1 atm and 300 K; Fig. 1*B*).

When inserted into the POPC bilayer, the secondary structure of both APP NMR structures fluctuates only marginally and preserves an α-helical conformation. The TM domain of APP_2LLM_ remains close to the initial NMR structure (root mean square deviation, 1.9 ± 0.2 Å). The important bend observed in the APP_2LP1_ initial structure straightens within the first 20 ns of simulation and remains straight throughout the rest of the simulation (>300 ns; supplemental Table S1). The final structures for the TM domain, starting from both initial conformations, are nearly identical (root mean square deviation, 1.3 ± 0.1 Å) and are characterized by a straight TM α-helix with a small kink at the paired residues Gly^708^-Gly^709^ (Fig. 1*B*). The APP_2LLM_ TM helix tilts slightly less with respect to the membrane surface compared with APP_2LP1_ (∼20 ± 7 and ∼36 ± 5°, respectively). We also observe a different behavior of the N terminus domain. Consistent with the NMR observations and theoretical models, the APP_2LP1_ N terminus amphiphilic helix (Gln^686^–Asn^698^) lies on the membrane surface, and a lysine belt (Lys^687^, Lys^699^, and Lys^724–726^) anchors the TM and N terminus helices, respectively, in and onto the membrane. In the APP_2LLM_, an extra turn forms at the N terminus of the TM domain (Ser^697^–Lys^699^), hindering the interaction of the N terminus helix with the membrane surface. This is, however, consistent with the NMR ensembles, where this terminal helix is flexible and explored a broad spectrum of conformation (angles ranging from 75 to 180° with respect to the TM domain).

The water molecules mainly interact with residues close to the membrane interface (Gly^700^, Ala^701^, Leu^720^, and Leu^721^), and poor solvation is observed in the core of the TM domain, where water molecules interact with Gly^708^ or Gly^709^ (Fig. 1*C*). The water molecules access the TM core from the N terminus by interacting with the ^700^G*XXX*G*XXX*G^708^ motif or from the C terminus by interacting with the residue Thr^719^ side chain (supplemental Fig. S1).

To characterize the free energy landscape associated with the helix bending in a membrane bilayer, we explored the conformational space associated with the two dihedral angles ϕ and ψ, respectively, connecting the backbone nitrogen atoms of Gly^708^ to Gly^709^ and Gly^709^ to Val^710^. Consistent with the unbiased MD simulations, the metadynamics trajectories showed that the POPC bilayer strongly confines the APP-TM structure to a well defined straight helical conformation (minimum at [−35°; −43.5°]; Fig. 1*D*). The free energy penalty needed to explore bent conformations similar to the APP_2LP1_ NMR structures is as high as 6–7 kcal/mol, suggesting that in the membrane environment, a straight conformation is more populated than the curved one.

##### The APP-TM Domain Explores α-Helical Kinked Conformations in Detergent Micelles

Because both NMR structural ensembles were solved in detergent micelles and not in a lipid bilayer, we used MD simulations to study the behavior of APP when embedded in micelles having properties mimicking the experimental setup. The APP_2LP1_ NMR ensemble was used as starting structures to investigate whether its tertiary structure could be stabilized by the micelle. The micelles inscribing the APP_2LP1_ and APP_2LLM_ NMR structures are reported to have different sizes (Fig. 1*A*) because of the different lipid detergents used in the experiments. Two systems containing the juxtamembrane and TM helices (residues Val^683^–Tyr^728^) embedded into different micelles were therefore assembled. As described by Barrett *et al.* (11), a ∼35 Å diameter micelle was considered for the first system (M35), whereas a larger micelle (*i.e.*, ∼45 Å diameter) was used in the second system (M45) to study the dependence of the APP conformation on micelle size. Each micelle was first equilibrated separately in a water box. The micelle assembly converged and remained stable after 50 ns. The protein was then inserted into the micelle, and the simulations were extended for at least an extra 150 ns. Because the NMR spectra were measured at 45 °C, we performed simulations under physiological conditions (300 K) and at a higher temperature (318 K) consistent with the experimental setup (supplemental Table S1).

As shown in Fig. 2, the M35 micelle severely affects the structure of APP. The size of this micelle appears too small to fully accommodate the TM helix, thus exposing the helical interface that encloses the ^700^G*XXX*G*XXX*G^708^ motif to the solvent (Fig. 2*A*). The Thr^714^ and Thr^719^ hydroxyl groups and the Gly^700^, Gly^704^, Gly^708^, and Val^711^ backbone atoms participate the most in the interaction with water molecules (Fig. 2*B*). The TM helix is highly curved, kinking either between residues Gly^708^ and Gly^709^, which is consistent with the NMR, or more rarely between residues Gly^709^ and Val^710^. The larger M45 micelle can better accommodate the TM domain (Fig. 2*A*). The structure alternates between a curved and straight structure, with a preference for the latter one. Similar to M35, but to a lesser extent, the threonine side chains and glycine backbone interact with water molecules (Fig. 2*B*). The solvation of the TM domain therefore seems to be the major driving force producing a kink in the APP-TM domain. Because only one interface is exposed to the solvent, solvation is observed with the same periodicity as the helical motif (Fig. 2*B*). Consistent with the NMR structure, the amphiphilic N terminus helix lies on the surface of the micelles. Heating the system to 318 K, as in the experimental setup, only marginally affected the APP structure. The ensemble of conformers remained comparable to the one at 300 K (population root mean square deviation, 2.9 ± 0.7 and 3.0 ± 0.4 Å, respectively).

The NMR APP_2LP1_ structural ensemble, however, is characterized by a single conformation of the TM backbone. To explain the discrepancy between our observations and the NMR results, we decided to back-compute the RDCs. The RDCs provide long range structural information and can be used to estimate the fold of proteins. They have been shown to be in good agreement with the results of MD simulations (33). Because the exact characteristics and composition of the NMR sample cannot be defined, we used a genetic algorithm to select an ensemble of conformers that would best fit the experimental RDCs. A set of 3000 structures was first extracted from the MD simulations performed in both M35 and M45. For each structure, the backbone N–H vector was defined, and the alignment tensor was computed using singular value decomposition. Based on several runs of the genetic algorithm, on the average, a set of 500 structures was able to best fit the experimental RDCs. The correlation of these back-calculated RDCs extracted from the MD trajectories was comparable to the one calculated for the NMR ensemble (Q factor 0.23 and 0.31, respectively, supplemental Fig. S2*a*). The selected ensemble was finally composed by 33 structures that showed a mixed population of straight and bent helices extracted from the M35 and M45 simulations (supplemental Fig. S2*b*). Similar results were obtained when considering separately the M35 and M45 systems. This provided a solid indication that APP-TM is able to explore a larger and more heterogeneous set of conformations in the micelle environment, as documented by MD simulations.

Finally, metadynamics simulations in M35 and M45 micelles were used to more exhaustively explore the conformational space of APP (Fig. 2*C*). The free energy landscape for both micelles validated the unbiased MD simulations where the structures inserted into a micelle could freely explore the bent and straight conformations on a multinanosecond time scale. The barrier to pass from the straight helix minimum (slightly more populated) to a bent structure is in fact in the order of ∼3 kcal/mol, and it is thus accessible via thermal fluctuations (Fig. 2*C* and supplemental Fig. S3). Consistent with our simulations, the conformational space associated with this transition is expected to be sampled on a 10^2^ ns time scale.

##### The APP-TM Responds to Structural Perturbations by Exploiting Its Native Flexibility

EPR power saturation methods can be used to measure the membrane depth of a protein. By monitoring the collision rate of a spin label with a paramagnetic relaxation agent, the solvent accessibility of the label can be estimated (Fig. 3*A*). MTSL is a commonly used nitroxide spin label, covalently bonded to genetically engineered cysteines in the protein (Fig. 3*B*). The depth parameter is determined by the ratio of the collision rate of two different probes (*e.g.*, oxygen and Cr(C_2_O_4_)^3−^) with the label and can be defined as a function of the distance of the label from the phosphate group (34, 35). EPR DEER has become a popular method for measuring the distance distributions between paramagnetic spin labels in proteins (36–38) when two MTSL labels are bonded to cysteines (Fig. 3*C*). The DEER technique has the advantage that it neither requires the crystallization of the protein complex, nor is it limited by the complex size. Distances in the range of 18 to 60 Å can be measured in biomolecules (39–42).

We used the EPR data reported by Barrett *et al.* (11) to clarify and rationalize our MD observations for the APP structure in the membrane bilayer. We also assessed the impact on the tertiary structure of several point mutations in the APP-TM. Τwo mutation sites (Gly^700^ and Leu^723^) used for the insertion of the MTSL spin labels in the DEER EPR were considered. First the single and the double mutants (G700C, L723C, and G700C/L723C) were inserted into a POPC membrane and simulated under physiological conditions. The mutation on the Gly^700^ site significantly affected the structure and the dynamics of the APP. The position of the N terminus helix on the membrane significantly differed from the wild type (supplemental Fig. S4). This mutation changes the flexibility of the loop connecting the juxtamembrane helix to the TM domain. The solvation of the APP-TM core is therefore also affected (supplemental Fig. S5). In contrast, the L723C mutation did not significantly alter the structure of the APP. The tilting and position of the juxtamembrane helix remained similar to the wild type. The double mutant G700C/L723C mainly recapitulated the features observed for the single G700C system.

For all three mutants, we attached a MTSL spin label to each cysteine (G700CM, L723CM, and G700CM/L723CM). Again, the Gly^700^ single and Gly^700^/Leu^723^ double mutations perturb the APP structure the most, with the TM helix remaining strongly curved and the N terminus helix rearranging itself on the top of the membrane. The helix started to slowly straighten after 130 ns of simulation (supplemental Fig. S4). The G700CM/L723CM system was therefore extended to 300 ns, during which time the helix remained fully straight. As observed previously, the L723CM single mutant only marginally affected the APP structure, with the TM domain remaining straight, as in the wild type conformation.

The depth parameter of APP inserted in the lipid bilayer was computed for the straight and curved structures of the G700CM/L723CM mutant, respectively (Fig. 3, *A* and *B*). A representative population composed of 500 structures (over 50 ns) was extracted for each conformation. The power saturation profile of the straight structure better matches the experimental values than the curved one (correlation coefficient, 0.96 and 0.88, respectively). Consistent with the experimental data, the most buried residues are located at around Val^711^ for the straight structure. This maximum shifts from Val^711^ to Ile^716^ for the bent structure. The profile of the bent helix for the flexible domain (Gly^700^–Gly^709^) better matches the experimental values. However, as observed during simulations, this region is easily perturbed by the experimental protocol and bends when spin labels are attached.

The computed distance distribution calculated using three different DEER prediction algorithms (MMM, mtsslWizard (24), and Pronox (25)) were compared for the straight and curved helical conformations extracted from MD simulations. All three algorithms predicted a similar distance, and on average, the distances were 31.3 ± 3.7 Å for a bent helix and 38.9 ± 4.4 Å for the straight helix (Fig. 3*C* and supplemental Table S2). When compared with the experimental distribution, these predictions support a more curved helical conformation. However, the straight conformation cannot be excluded, because these algorithms are parameterized to predict the distance of proteins in solution (36). The presence of the membrane limits the flexibility of the probes and, most importantly, can strongly trap their hydrophobic regions as seen in MD (Fig. 3*B*). We therefore decided to extract the position of the N–O bond from the MTSL tags during MD simulations and compute the corresponding distance distribution (Figs. 3*D* and supplemental Fig. S6). As expected, the distributions systematically shift toward shorter distances in comparison to DEER prediction algorithms. This difference is well pronounced for the straight helix distribution, becoming comparable to the bent helix distribution. Because of the hydrophobic nature of the spin labels, they are buried deep into the membrane, thus limiting the conformational space of their rotational degrees of freedom (supplemental Fig. S7). Furthermore, the presence of this anchor allows the TM helix to explore a bent conformation in the membrane bilayer.

Metadynamics also confirmed that, by adding MTSL labels to APP at positions 700 and 723, the structure acquired an increased flexibility compared with the wild type and was able to explore more curved structures, as was observed in the micelle environment. A straight conformer is, however, slightly more preferred by ∼1 kcal/mol over a bent conformation (Fig. 3*D*). This is consistent with the observation in free dynamics, where the bent helix straightens. Because the ϕ dihedral angle explores more freely its conformational space, Gly^708^ may confer more flexibility to the APP-TM domain. Based on the free energy landscape, we built an ensemble of conformers that follow a Boltzmann distribution. The distance distribution computed on this ensemble allows for better accounting for the broad distribution observed experimentally (Fig. 3*C*) and strongly hints to a heterogeneous population alternating between straight and bent APP-TM conformations, when spin labels are attached.

##### Changes in APP-TM Flexibility and Hydration Affect γ-Secretase Cleavage

In MD we clearly observed that position 700 is crucial for modulating the overall flexibility of APP in a membrane bilayer. The peculiar location of Gly^700^ in the loop connecting the juxtamembrane helix and the TM segment is key to affecting the native flexibility of the ^700^G*XXX*G*XXX*G^708^ motif. Thus, to gain further mechanistic details about the role of Gly^700^, we generated a series of point mutations and studied their effect on APP structure, as well as on the processing by purified γ-secretase. To determine the role of Gly^700^, we gradually increased the side chain size producing G700A, G700L, and G700W species. The tryptophan mutant closely resembles the MTSL spin label with its bulky and amphipathic properties.

After expression and purification of the recombinant wild type and mutated APP-C100His substrates, we performed *in vitro* activity assays with all the constructs. In this enzymatic reaction, γ-secretase cleaves APP-C100 into different Aβ (N-terminal) and AICD (C-terminal) peptides, in the presence of a mixture of phosphatidylcholine and phosphatidylethanolamine, closely mimicking a neuronal membrane environment. Following the activity test, we analyzed the resulting products by Western blotting, immunoprecipitation combined with MALDI-TOF mass spectrometry (IP/MS), or ELISA. First, our results clearly demonstrate that both wild type and mutated proteins are processed by the γ-secretase complex *in vitro* to generate Aβ or AICD cleavage products (supplemental Fig. S8*a*). To gain further insight into how these mutations can modulate the relative profiles of different Aβ and AICD products, we analyzed the same samples by IP/MS (Figs. 4 and supplemental Fig. S8*b*) and quantified both Aβ38 and Aβ40 production by ELISA (supplemental Fig. S8*c*). Noteworthy are the distinct Aβ cleavage profiles observed for wild type and mutated substrates. As shown in Fig. 4*B*, and as previously observed by others (6, 43), the G700A mutation caused an increase in the production of Aβ38 when compared with Aβ40, with only a limited effect on the longer, more pathogenic Aβ42 and Aβ43. As estimated by ELISA, this mutation caused a significant ∼2-fold increase in the Aβ38/Aβ40 ratio, when compared with the wild type substrate (supplemental Fig. S8*c*). The same trend was observed for both G700L and G700W, with an even larger Aβ38 production causing a ∼2.7-fold increase in the Aβ38/Aβ40 ratio (supplemental Fig. S8*c*). This behavior partially reflects the phenotype of small chemical γ-secretase modulators on Aβ generation, which shift Aβ production toward shorter and less fibrinogenic Aβ38 (44). In contrast to the mutations tested here, γ-secretase modulators also reduce Aβ42 production (44). Gly^700^ substitutions had no significant effect on the ϵ-cleavage site because the two AICD products (AICD50 and AICD51) remained close to equimolar ratio (supplemental Fig. S8*b*). These results therefore support the view that structural changes in APP-G700 mutants translate in altered cleavage at the γ-site, but not at the ϵ-site.

**FIGURE 5.**
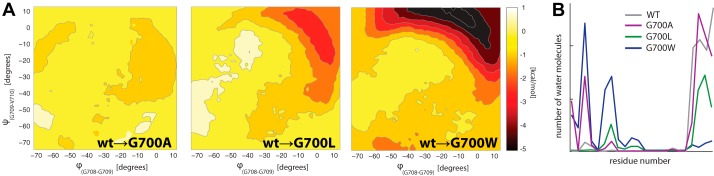
**Flexibility and hydration of APP Gly^700^ mutants in a membrane bilayer.** *A*, difference in the free energy landscape between wild type and APP mutants (G700A, G700L, and G700W) associated to ϕ and ψ dihedral angles connecting the nitrogen atoms of Gly^708^ to Gly^709^ and Gly^709^ to Val^710^, respectively, as calculated by metadynamics MD (see supplemental Table S1). *B*, the distribution of the water molecules solvating the TM-APP helix is reported for the wild type and APP mutants.

This increase of the Aβ38/Aβ40 ratio can be rationalized at a molecular level using the results from MD simulations of these Gly^700^ mutants. Indeed, the size and nature of mutant residues at position 700 seem to have a major influence on the flexibility and hydration of the APP-TM domain. In the APP-TM domain, they invariantly increase the native flexibility, thus exploring, as seen in metadynamics simulations, more easily conformations that deviate from the straight helical structure of the wild type (Fig. 5*A*), This is also observed in MD simulations in micelles and G700CM and G700CM/L723CM species (supplemental Fig. S4). This is also reflected in the general increase of the kinked APP-TM population, which passes from an average 169 ± 6° in G700A to 162 ± 9° in G700L and 128 ± 8° in G700W (supplemental Fig. S4). In particular, G700A, which is a less bulky and hydrophobic substitution than G700W, accordingly produces a conformation more similar to the wild type in the membrane. However, G700A produces larger hydration of the ^704^G*XXX*G^708^ motif than in the wild type (Fig. 5*B*), which probably contributes to facilitate γ-secretase cleavage at the Aβ38 γ-site. In G700L, both flexibility and hydration continue to increase with respect to the wild type and G700A, whereas G700W produced the more relevant changes in these two properties (Fig. 5, *A* and *B*, and supplemental Fig. S4). This reinforces the hypothesis that mutation at position 700 has the ability to modulate the flexibility and hydration of APP in the membrane, thus affecting its presentation to γ-secretase and the relative cleavage profile, which appears systematically shifted toward a larger production of Aβ38 peptides.

## DISCUSSION

The alteration in the proteolytic cleavage of the APP-TM domain has been linked to the pathogenesis of Alzheimer disease. The absence of direct structural information on the APP/γ-secretase complex currently hinders a complete understanding of the enzymatic mechanism and its pathogenic implications. Recently, however, two structures of the APP-TM domain were solved using NMR, thus providing the possibility to explore further the native properties of APP. In the first structural ensemble, the APP-TM is mainly defined by a straight α-helical conformation, whereas an important bend was observed at residues Gly^708^-Gly^709^ for the second reported NMR ensemble. Because these structural ensembles have been solved in detergent micelles, they are not fully representative of the APP embedded in its natural environment. In this study, we used molecular dynamics simulations coupled to available NMR and EPR data to dissect the structural and dynamical properties of the TM domain of APP. We found that once inserted into a lipid bilayer representative of the neuronal synaptic envelope, the TM domain of the APP mainly adopts a straight α-helix conformation. These results are in agreement with previous MD simulations based on models of the TM domain alone (12). The TM domain is characterized by a dynamic structure, in which the paired residues Gly^708^-Gly^709^ confer some flexibility to the TM domain and allow it to adapt to the fluctuations of the membrane thickness.

The experimental conditions used to characterize the APP-TM structure are at the origin of the discrepancy between the structure of APP in a bilayer and in micelles. The driving force for the formation of the bend is the solvation of the ^700^G*XXX*G*XXX*G^708^ motif, which is largely exposed to the bulk solvent in small micelles. Importantly, although less abundant, hydration is also present for APP in the membrane bilayer, where Gly^704^ is the most hydrated residue, but likewise residues at the γ- and ϵ-cleavage sites (*i.e.*, Gly^708^ and Val^711^) are visited by water molecules (Fig. 1*C*). This additional property of the ^700^G*XXX*G*XXX*G^708^ motif might be relevant for the modulation of γ-secretase cleavage profile *in vivo*.

Because different surfactants (*e.g.*, dodecylphosphocholine and LMPG) composed the micelles used in NMR spectroscopy, the APP_2LLM_ structure was probably embedded into larger micelles, producing a much straighter conformation. The exact characteristics of the NMR samples, however, are difficult to determine, and back-calculated RDCs from MD conformations showed that a heterogeneous population of straight and bent α-helices is able to better fit the experimental data. This suggests that the APP-TM backbone can adopt several different conformations under the effect of the lipid environment. These results are consistent with the free energy landscape of APP in micelles, where we observed that APP could explore almost freely the conformational space associated with the TM bending.

To give further support to our model, we compared it with the EPR power saturation and DEER experiments used to analyze the structural characteristics of APP-C99 in the POPC liposome (11). Based on the MD simulations performed in the POPC membrane, a straight TM α-helix model better matches the reported EPR power saturation profile (Fig. 3*A*). By enforcing a bend as observed in the APP_2LP1_ structure, the most buried amino acid shifted by ∼5 residues. This further validates the previous results of a straight TM domain in the biologically relevant environment.

Page *et al.* (43) and Munter *et al.* (6) have shown that mutations in the ^700^G*XXX*G*XXX*G^708^ motif affect the cleavage by γ-secretase. In particular, the Gly^700^ mutation may not only perturb the recognition by γ-secretase but drastically modifies the dynamic properties of APP-TM. Gly^700^ is the last residue of the loop connecting the juxtamembrane domain to the TM domain. Therefore, perturbing this region directly affects the APP backbone flexibility, as clearly seen here in all the mutations perturbing this position (*i.e.*, G700A, G700L, G700W, and G700CM), and causes TM kinking and the juxtamembrane domain to rearrange itself (Figs. 5*A* and supplemental Fig. S4). Moreover, we observed that this effect is associated with a significant increase of the hydration of the APP-TM N terminus, which is evident already for conservative mutations, such as G700A (Figs. 1*C* and 5*B*). Therefore, the combined perturbation by point mutations of both the flexibility and hydration of the ^704^G*XXX*G^708^ motif directly affects the γ-secretase cleavage profile, favoring in turn Aβ38 production (Fig. 4 and supplemental Fig. S8).

Moreover, when MTSL spin labels are bound to G700C mutants, we found that the dynamical properties of the TM domain are even more dramatically affected. The curved and straight conformations of the TM helix were observed in the POPC bilayer, with a slight preference for the latter one. The deep burial of the MTSL spin label into the membrane surface forces the APP helix to bend. The MTSL spin labels have shown a preference for hydrophobic cavities (36, 45). In the presence of a membrane, these markers have a tendency to bury themselves into the hydrophobic core of the membrane (Fig. 3*B* and supplemental Fig. S7). The differences observed between the predicted distance distribution and the one extracted from MD simulations could be explained by the fact that the rotameric libraries used in MTSL prediction algorithms are based on simulations in solution (36). They are therefore not able to consider the effect of the hydrophobic environment of the membrane. Once buried into the membrane, the spin labels can no longer explore the full conformation space, biasing the expected simulated distance toward shorter distances. The broad distance distribution observed experimentally is thus caused by the simultaneous presence of the straight and kinked helices, as documented by a Boltzmann-weighted ensemble of structures, which matched more accurately the experimental results (Fig. 3*C*). Consistent with experimental findings, the free energy landscape also confirmed that Gly^708^ confers more flexibility to the APP-TM (11). By mutating this residue, the flexibility is reduced, thereby biasing the structure toward a straighter helix. This produces a sharper DEER distance distribution.

APP appears to have a highly dynamic structure that can easily respond to changes in the lipid environment and to point mutations. In this context, the atomistic interpretation of experimental data accessible by integrative dynamic modeling strategies (46) coupled to enhanced sampling molecular dynamics simulations was fundamental in discovering how APP, although having preferential conformers under different conditions, is more properly described by a dynamic ensemble of conformations that explore the intrinsic flexibility encoded in the Gly^708^-Gly^709^ motif. Alterations in the membrane composition, especially the concentration of cholesterol, have been linked to Alzheimer disease. It is hypothesized that they affect Aβ aggregation into amyloid plaques (47–49). Because a change in the concentration of cholesterol would affect the flexibility and thickness of the membrane (50), the structure of the APP-TM domain would probably also be affected.

We have recently shown that familial Alzheimer disease-causing mutations located in between the γ- and ϵ-cleavage sites and altering the cleavage profile by increasing the production of fibrilogenic Aβ42 seem not to affect the flexibility of the TM domain but rather its presentation to γ-secretase (44). We demonstrated in this study how mutations perturbing position 700, well above the γ-site, systematically shift Aβ production from Aβ40 toward shorter and less fibrillogenic Aβ38. Based on these observations, it is tempting to suggest that one can modulate the production of Aβ peptides of different length by selectively targeting APP above or below the Gly^708^-Gly^709^ motif. Consistent with this notion, we observed that the effect of mutations at the Gly^700^ site partially reflect the phenotype of small chemical γ-secretase modulators with the increase of Aβ38 peptides (44). A possible explaination for this similarity can be found on the molecular mechanism of modulation of APP flexibility, which proposes γ-secretase modulators equally trigger the formation of a kink in APP at the Gly^708^-Gly^709^ motif.

Finally, the low resolution cryo-EM map of γ-secretase suggests a possible arrangement of the enzymatic binding site, which might accommodate the APP substrate (23, 51). Given the poor resolution, either a curved conformation as for the APP_2LP1_ structure observed in micelles or a straight α-helical structure mainly present in the membrane bilayer could be bound to the γ-secretase active site (11). Thus, the role of the observed induced flexibility of APP can be 2-fold when forming the enzymatic reactants complex with γ-secretase: (i) the modulation of TM kinking would be functional for better exposing specific cleavage sites to the γ-secretase, and (ii) the recruitment of water molecules would facilitate catalytic cleavage inside the membrane.

## Supplementary Material

Supplemental Data

## Footnotes

2 The abbreviations used are:

Aβ: amyloid-β
APP: amyloid precursor protein
TM: transmembrane
AICD: APP intracellular domain
EPR: electron paramagnetic resonance
DEER: double electron-electron resonance
MD: molecular dynamics
LMPG: lysomyristoylphosphatidyl glycerol
MTSL: methanethiosulfonate
POPC: palmitoyloleoly phosphatidylcholine
CM: cysteine bound to MTSL
RDC: residual dipolar coupling
CHAPSO: 3-[(3-cholamidopropyl)dimethylammonio]-2-hydroxy-1-propanesulfonic acid
EPR: electron paramagnetic resonance
IP/MS: immunoprecipitation combined with MALDI-TOF mass spectrometry.
