# Supplementary material for: Perturbations of the Straight Transmembrane α-Helical Structure of the Amyloid Precursor Protein Affect Its Processing by γ-Secretase
Source: J Biol Chem. 2014 Jan 27;289(10):6763–74. doi: 10.1074/jbc.M113.470781 (PMC3945338; doi:10.1074/jbc.M113.470781)
Supplement: Supplemental Data [file supp_289_10_6763__index.html]

Perturbations of the Straight Transmembrane α-Helical Structure of the Amyloid Precursor Protein Affect its Processing by γ-Secretase — Perturbations of the Straight Transmembrane α-Helical Structure of the Amyloid Precursor Protein Affect Its Processing by γ-Secretase — The Flexibility of APP Modulates Its Proteolytic Processing — Supplemental Data 

# Perturbations of the Straight Transmembrane α-Helical Structure of the Amyloid Precursor Protein Affect Its Processing by γ-Secretase

## Supplemental Data

**Files in this Data Supplement:**

- supplemental data (.pdf, 5.2 MB) - supplemental data
