# Supplementary material for: Perturbations of the Straight Transmembrane α-Helical Structure of the Amyloid Precursor Protein Affect Its Processing by γ-Secretase
Source: J Biol Chem. 2014 Jan 27;289(10):6763–74. doi: 10.1074/jbc.M113.470781 (PMC3945338; doi:10.1074/jbc.M113.470781)
Supplement: Supplemental Data [file supp_M113.470781_jbc.M113.470781-1.pdf]

Supporting Figures

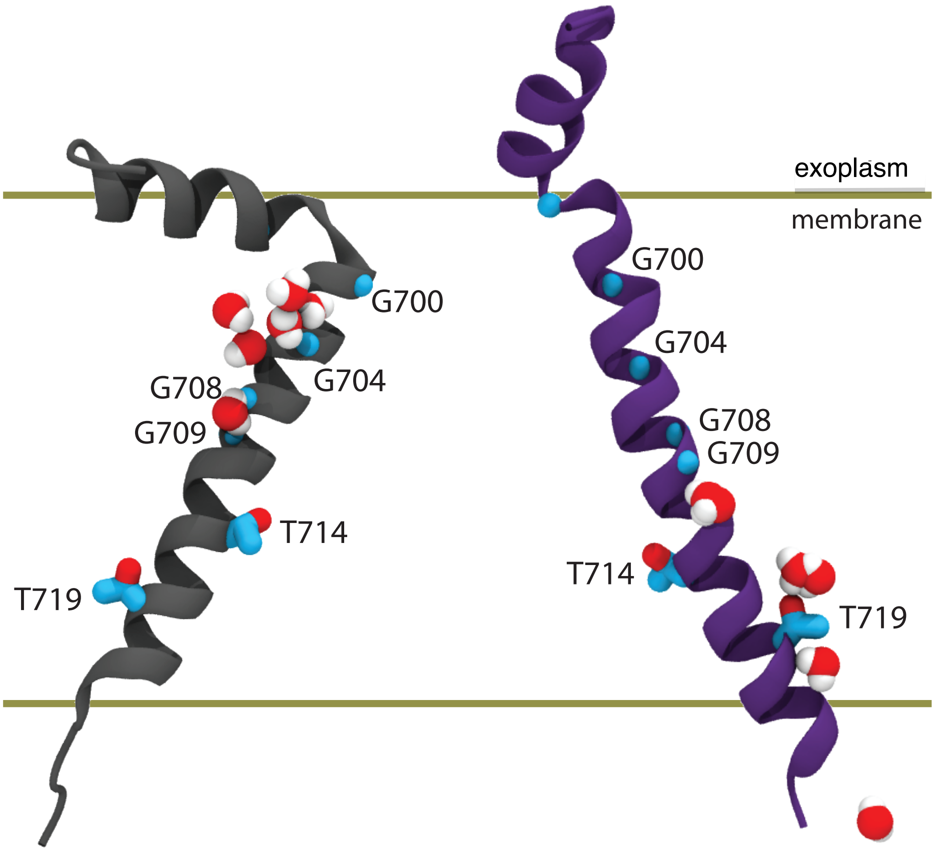

Figure S1: Solvation of the APP-TM. The water molecules access the TM core via the N-terminus (left) or C-terminus (right). The membrane surface is indicated with the gold lines. G700, G704 and G708 form the $G_{700}XXXG_{740}XXXG_{708}$ motif.

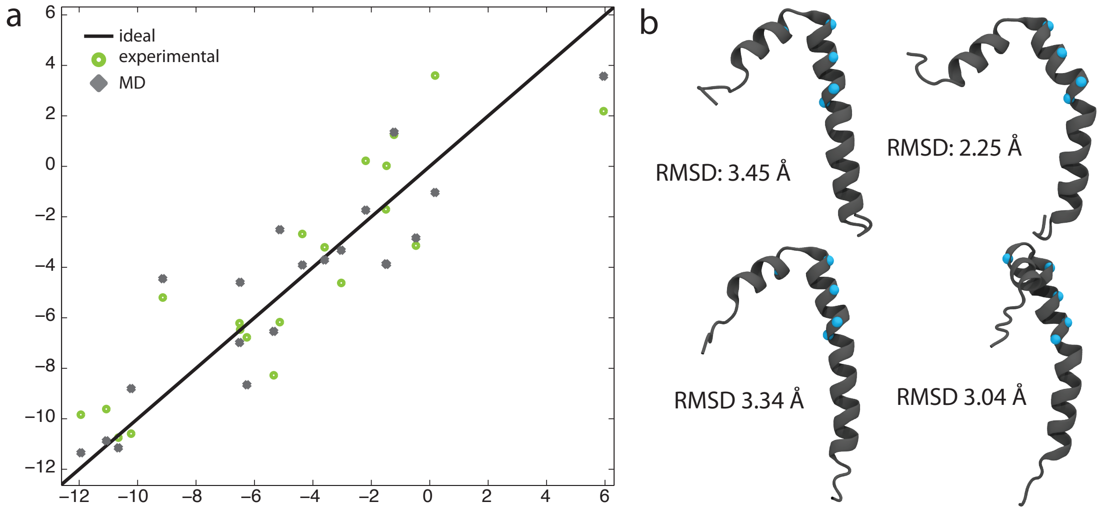

Figure S2: Residual dipole coupling analysis. (a) The correlation of residual dipole coupling (RDC) was back calculated from the NMR ensemble of structures (in green) and MD simulations performed in both micelles; M45 and M35 (in grey), and (b) The four major conformers are reported with their RMSD with respect to the APP_{2LP1} NMR structures.

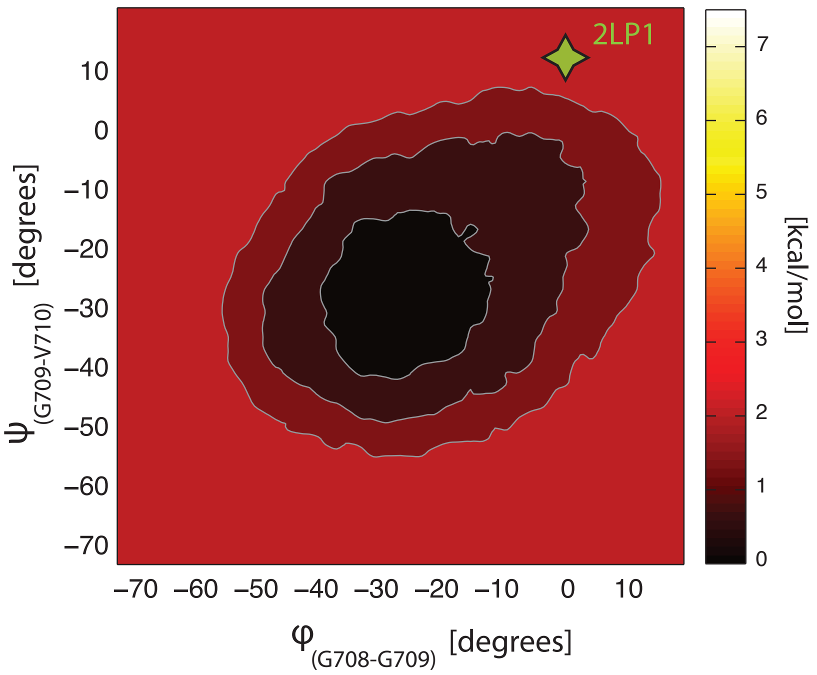

Figure S3: APP Free energy landscape in micelle. The free energy landscape associated to φ and ψ dihedral angles as calculated by metadynamics MD on the M35 system. The green asterisk indicates the average NMR conformation relative to the 2LP1 ensembles (see also Figure 2c).

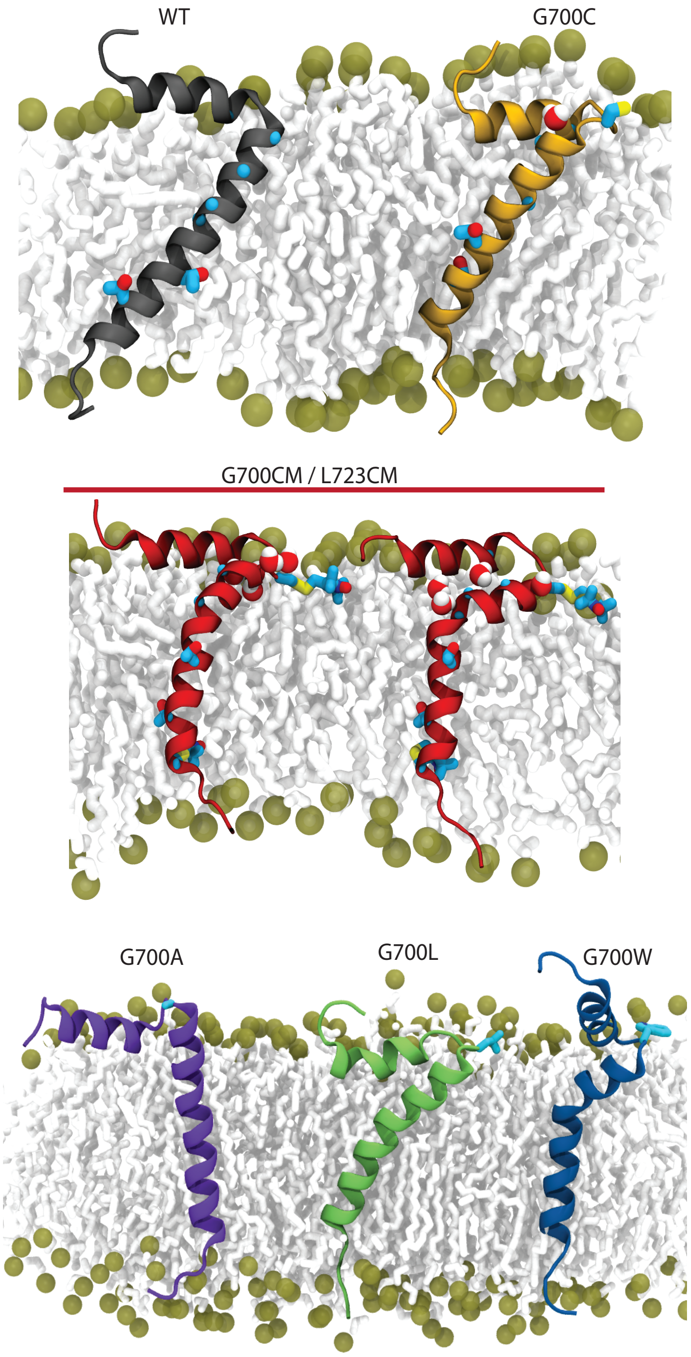

Figure S4: Structural models of APP mutants and spin labeled constructs. The wild type and relevant mutants, namely G700C, [G700CM/L723CM], G700A, G700L and G700W, were inserted in a POPC membrane bilayer and simulated with MD. Here selected snapshots from MD are reported (see Table S1).

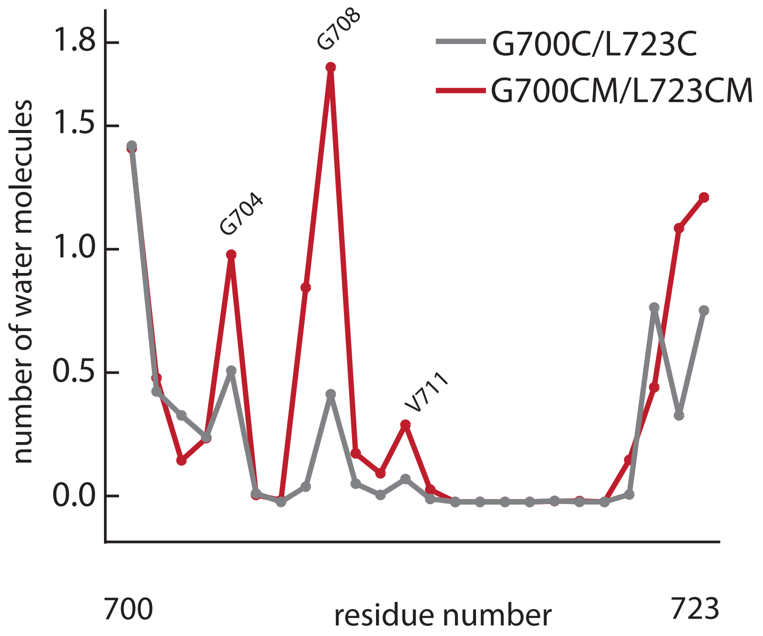

Figure S5: Solvation of G700C/L723C and G700CM/L723CM mutants. The relative level of solvation of APP-TM for G700/L723 mutations and spin-labeled constructs is reported (see also Figure 3c).

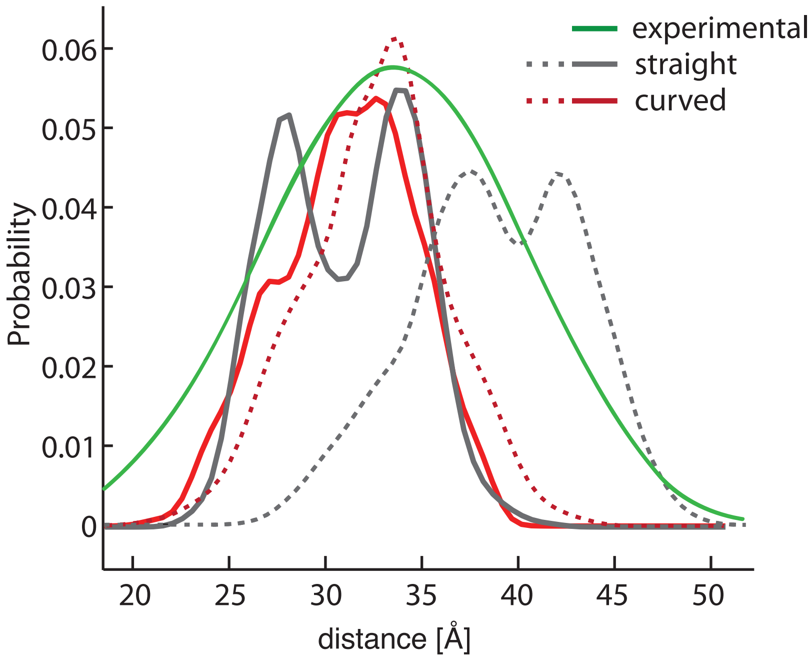

Figure S6: DEER distance distribution. The experimental distance distribution (in green) is compared to the predicted distance distribution using MMM (dashed line) and the distribution measured from MD with MTSL tags (solid line) (see Figure 3c,d).

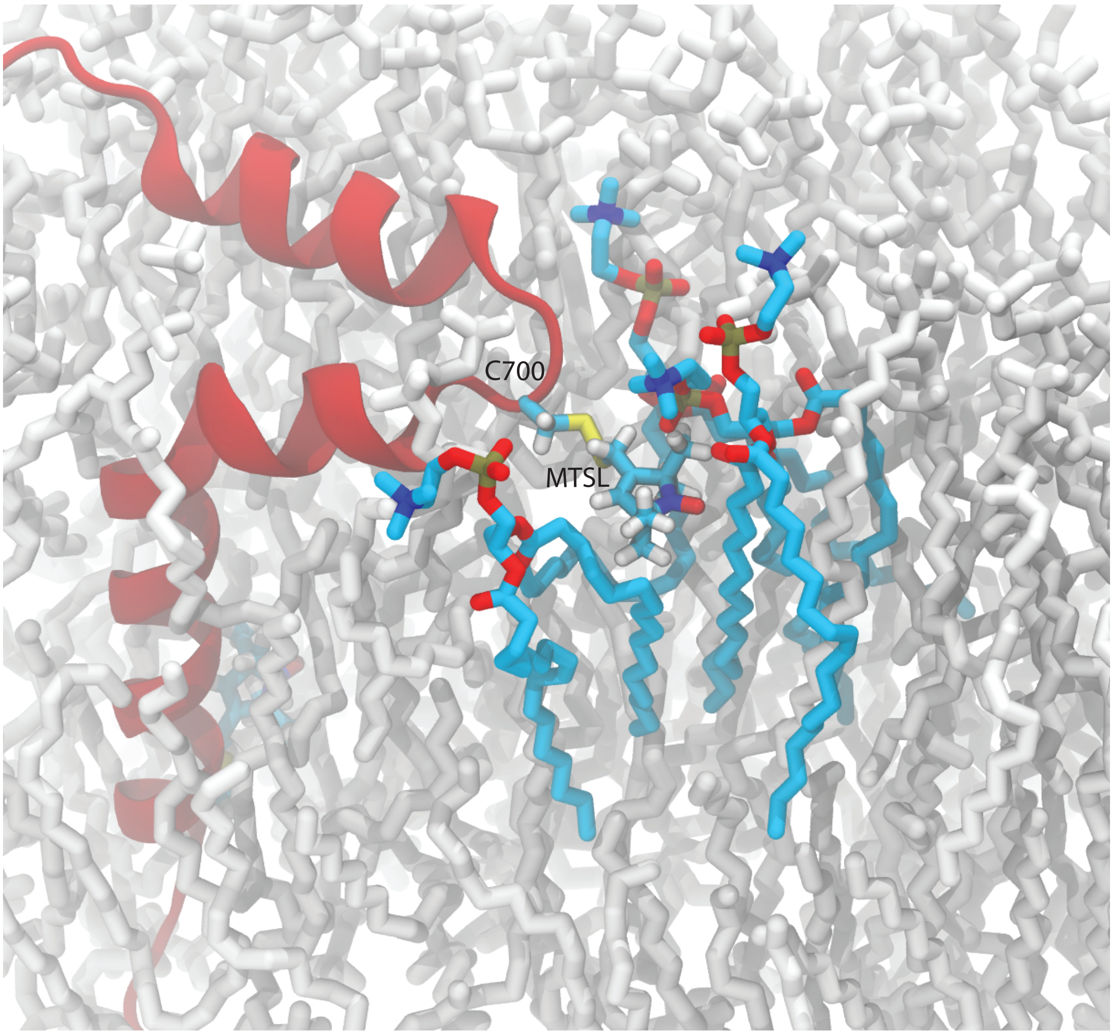

Figure S7: Spin labeled APP-TM domain. A snapshot from MD of the G700CM/L723CM system is reported. Lipids interacting with the MTSL spin label are highlighted in blue.

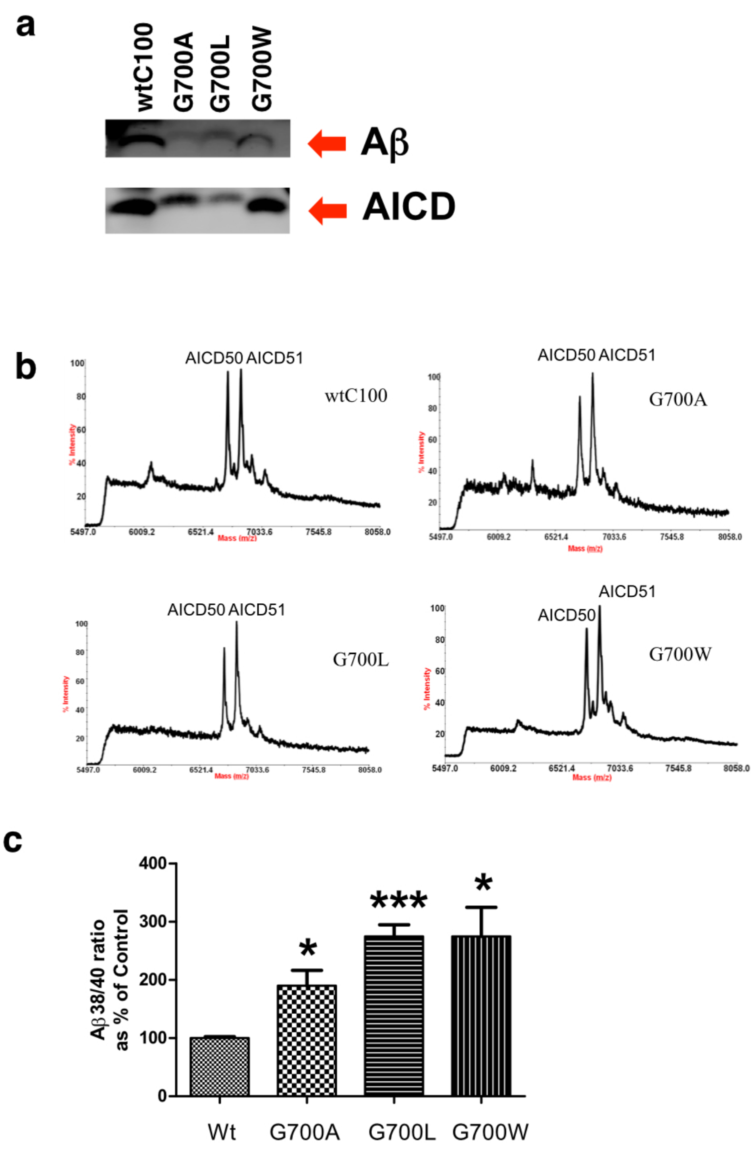

Figure S8: Proteolytic processing of APP-C100 wild-type and G700 mutants by highly purified γ-secretase. Recombinant wild-type (wt) and mutants G700A, G700L, G700W APP-C100 protein substrates were expressed in *E. coli* and purified on a Nickel-bound agarose resin. After protein normalization, substrates were incubated for 4 hours at 37°C with highly purified γ-secretase, and the cleavage products Aβs and AICDs were analyzed by Western blot (A), by mass spectrometry (B), or ELISA (C). (**A**) Western blot analysis confirms the processing of wt and mutated APP-CTFs *in vitro* by γ-secretase to generate Aβ (upper panel) and AICD (lower panel). (**B**) IP/MS profiles reveal that G700 mutations do not alter the profile of APP ε-site products AICD50 and AICD51, which remain in nearly equimolar ratio. These experiments were performed twice producing very comparable peak height values. (**C**) ELISA measurements reveal increased Aβ38/Aβ40 ratios for G700A, G700L and G700W mutants, when compared to the wt APP-C100 substrate. Student t-tests with two-tailed distribution are reported with * ($p<0.05$), ** ($p<0.01$), *** ($p<0.001$), and error bars representing standard errors (n=3).

Supporting Tables

Table S1. List of performed MD simulations

system name	lipid type	# of atoms	free MD	metadynamics
WT 2LP1	POPC	40,000	200 ns	100 ns
WT 2LLM	POPC	40,000	200 ns	-
M35	LMPG	65,000	150 ns	30 ns
M35 @ 318K	LMPG	65,000	100 ns	-
M45	LMPG	65,000	150 ns	30 ns
M45 @ 318K	LMPG	65,000	100 ns	-
G700C	POPC	40,000	100 ns	-
L723C	POPC	40,000	100 ns	-
G700C/L723C	POPC	40,000	120 ns	-
G700CM	POPC	40,000	180 ns	30 ns
L723CM	POPC	40,000	100 ns	-
G700CM/L723CM	POPC	40,000	300 ns	2 x 60 ns
G700A	POPC	40,000	200 ns	100 ns
G700L	POPC	40,000	200 ns	100 ns
G700W	POPC	40,000	200 ns	100 ns

Table S2. DEER distance predictions

system	MMM [Å]	MTSSLWizard [Å]	Pronox [Å]
straight helix	38.9 ± 4.4	40.61	39.9 ± 4.3
bent helix	31.3 ± 3.7	25.61	30.1 ± 4.3
